# Pyrazine Analogues Are Active Components of Wolf Urine That Induce Avoidance and Freezing Behaviours in Mice

**DOI:** 10.1371/journal.pone.0061753

**Published:** 2013-04-24

**Authors:** Kazumi Osada, Kenzo Kurihara, Hiroshi Izumi, Makoto Kashiwayanagi

**Affiliations:** 1 Division of Physiology, Department of Oral Biology, School of Dentistry, Health Sciences University of Hokkaido, Ishikari-Tobetsu, Hokkaido, Japan; 2 Aomori University, Kobata, Aomori, Japan; 3 Department of Sensory Physiology, Asahikawa Medical College, Asahikawa, Hokkaido, Japan; Utrecht University, The Netherlands

## Abstract

**Background:**

The common grey wolf (*Canis lupus*) is found throughout the entire Northern hemisphere and preys on many kinds of mammals. The urine of the wolf contains a number of volatile constituents that can potentially be used for predator–prey chemosignalling. Although wolf urine is put to practical use to keep rabbits, rodents, deer and so on at bay, we are unaware of any prior behavioural studies or chemical analyses regarding the fear-inducing impact of wolf urine on laboratory mice.

**Methodology/Principal Findings:**

Three wolf urine samples harvested at different times were used in this study. All of them induced stereotypical fear-associated behaviors (i.e., avoidance and freezing) in female mice. The levels of certain urinary volatiles varied widely among the samples. To identify the volatiles that provoked avoidance and freezing, behavioural, chemical, and immunohistochemical analyses were performed. One of the urine samples (sample C) had higher levels of 2,6-dimethylpyrazine (DMP), trimethylpyrazine (TMP), and 3-ethyl-2,5-dimethyl pyrazine (EDMP) compared with the other two urine samples (samples A and B). In addition, sample C induced avoidance and freezing behaviours more effectively than samples A and B. Moreover, only sample C led to pronounced expression of Fos-immunoreactive cells in the accessory olfactory bulb (AOB) of female mice. Freezing behaviour and Fos immunoreactivity were markedly enhanced when the mice were confronted with a mixture of purified DMP, TMP, and EDMP vs. any one pyrazine alone.

**Conclusions/Significance:**

The current results suggest that wolf urinary volatiles can engender aversive and fear-related responses in mice. Pyrazine analogues were identified as the predominant active components among these volatiles to induce avoidance and freezing behaviours via stimulation of the murine AOB.

## Introduction

The common grey wolf (*Canis lupus*) is found throughout the entire Northern hemisphere and preys on many kinds of mammals, including deer, rabbits, squirrels and mice. The wolf is a gregarious carnivore that has a more complex communication system than the comparatively solitary fox or cat [1]. The wolf utilizes visual, vocal and tactile cues, as well as chemo-olfactory modes of communication [1]. Urination provides a major mode of chemical communication for the wolf, and the way in that the urine is deposited relates to behaviours displayed during marking [2]. Urination and the chemical scent components of urine are intimately involved in communication, not only among individuals of the same species (conspecific communication), but probably also between individuals belonging to different species (interspecific communication).

Wolf urine induces avoidance behaviour in cattle [3], wild animals [4]–[7] and rats [8]. Wild animals frequently leave their natural surroundings and infiltrate into human habitats. As a practical matter, wolf urine is used to drive away these animals without killing them [4], [5], [7], [9]. Previous studies clearly indicate that wolf urine contains semiochemicals that repel their prey. Indeed, the urine of the wolf [10], coyote (*Canis latrans*) [11] and red fox (*Vulpes vulpes L.*) [12] all comprise sulphurous chemicals that emit a strong stench. Of these, Δ^3^-isopentenyl methyl sulphide [13] and its derivatives are candidate predator kairomones. However, their capacity to provoke avoidance behaviour is limited [9], [14]. Therefore, we hypothesized that wolf urine contains additional kairomones that are used in predator-prey chemosignalling.

The current study systematically analysed the avoidance behaviour [8], [15] and freezing behaviour [16]–[19] of female mice in response to wolf urine. We also employed gas chromatography-mass spectrometry (GC-MS) to characterize the volatile compounds in three different wolf urine samples. By comparing the results between the three samples, candidate fear-inducing pyrazine analogues were identified. Additional behavioural and immunohistochemical studies were performed to show that these candidate pyrazine analogues, and especially combinations thereof, encouraged significant freezing behaviour in mice, in part by the stimulation of the murine accessory olfactory bulb (AOB). These results support the proposition that wolf urine contains novel kairomones that mediate predator-prey chemosignalling. Notably, a cocktail of pyrazine analogues had a similar effect to that of the urine itself, suggesting that the cocktail may prove useful in applications to disperse wild animals.

## Results

### (a) Behavioural Studies

**Figure 1:**
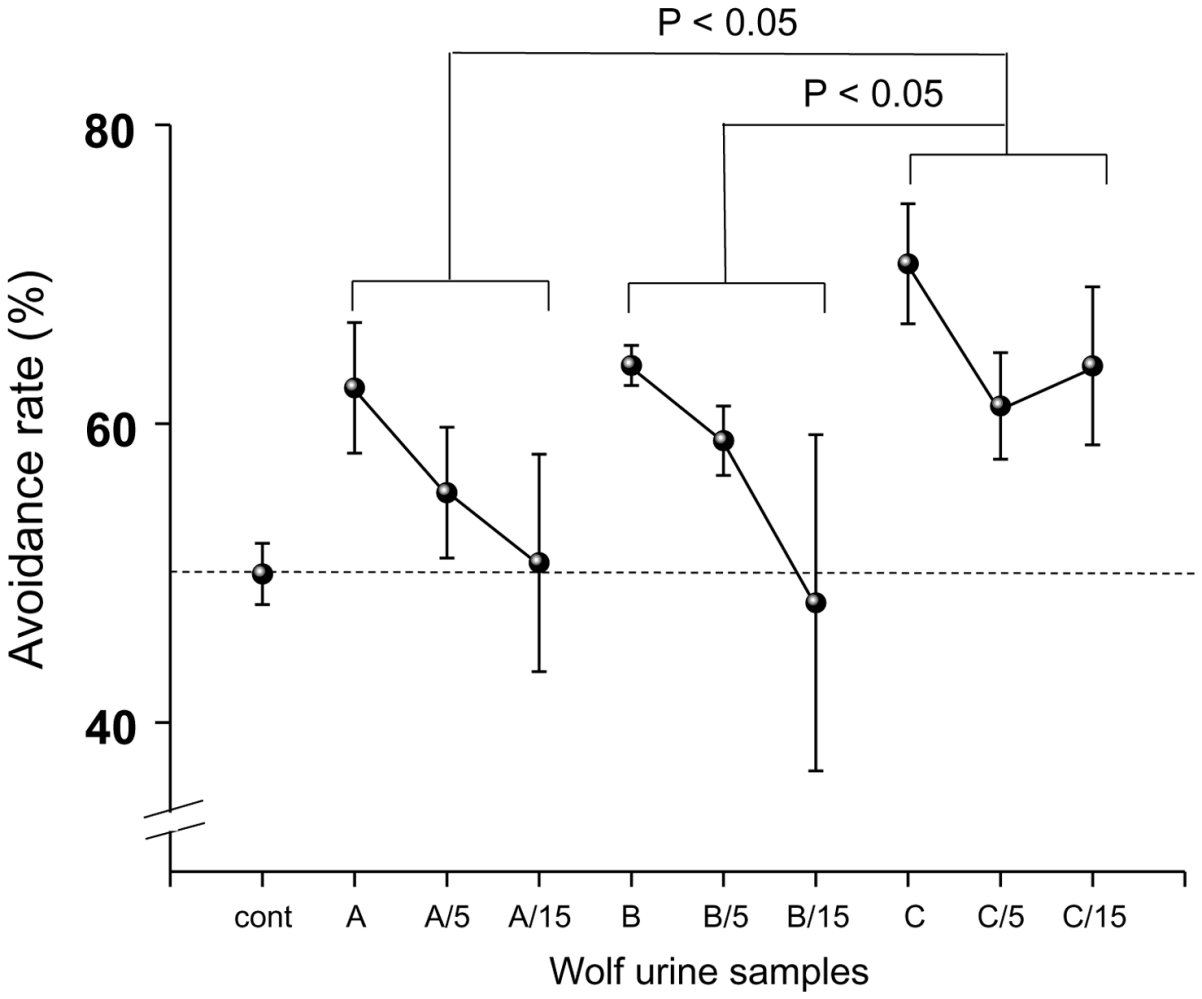
Avoidance rate during exposure of mice to wolf urine samples A, B and C. The avoidance rate was defined as the amount of time spent in the short arm of the Y maze with the control odour (water) divided by the total amount of time spent in both short arms with the wolf urine odour or the control odour. The statistical significance of the differences between the avoidance rates elicited by each of the wolf urine samples was assessed by repeated-measured ANOVA followed by Fisher’s PLSD post-hoc test.

We first conducted an avoidance test for the three wolf urine samples, A, B and C. These samples were harvested in November 2009 (sample A), January 2010 (Sample B) and March 2010 (sample C) (see details in the Materials and Methods section). Different concentrations of wolf urine (undiluted, 5-fold diluted and 15-fold diluted) were tested for their ability to induce avoidance behaviour in female mice (Figure 1). The average time spent in the short arm of the Y maze that contained either the wolf urine sample or the control odour (water) during the 3 min test varied between 100.7 sec and 128.4 sec. All three undiluted wolf urine samples elicited significant avoidance activity from the mice compared with the control (water vs. water) (sample A, 62.4±9.7, P<0.01; sample B, 63.9±3.0, P<0.01; sample C, 69.9±9.1, P<0.001 vs. control, 48.9±4.9; one-way analysis of variance (ANOVA) followed by Dunnett's post-hoc test. The entire data set for the three undiluted samples was subjected to repeated-measure ANOVA. This analysis revealed a significant main effect of the wolf urine samples on avoidance activity (F(3, 20) = 7.478, P<0.01). Fisher’s protected least squares difference (PLSD) post-hoc test indicated that the avoidance activity of sample C was higher than that of sample A (P<0.05) or sample B (P<0.05). Although the avoidance rate for all three samples tended to decrease with dilution, diluted sample C continued to demonstrate significantly higher aversive effects toward the mice than the other two urine samples.

**Figure 2:**
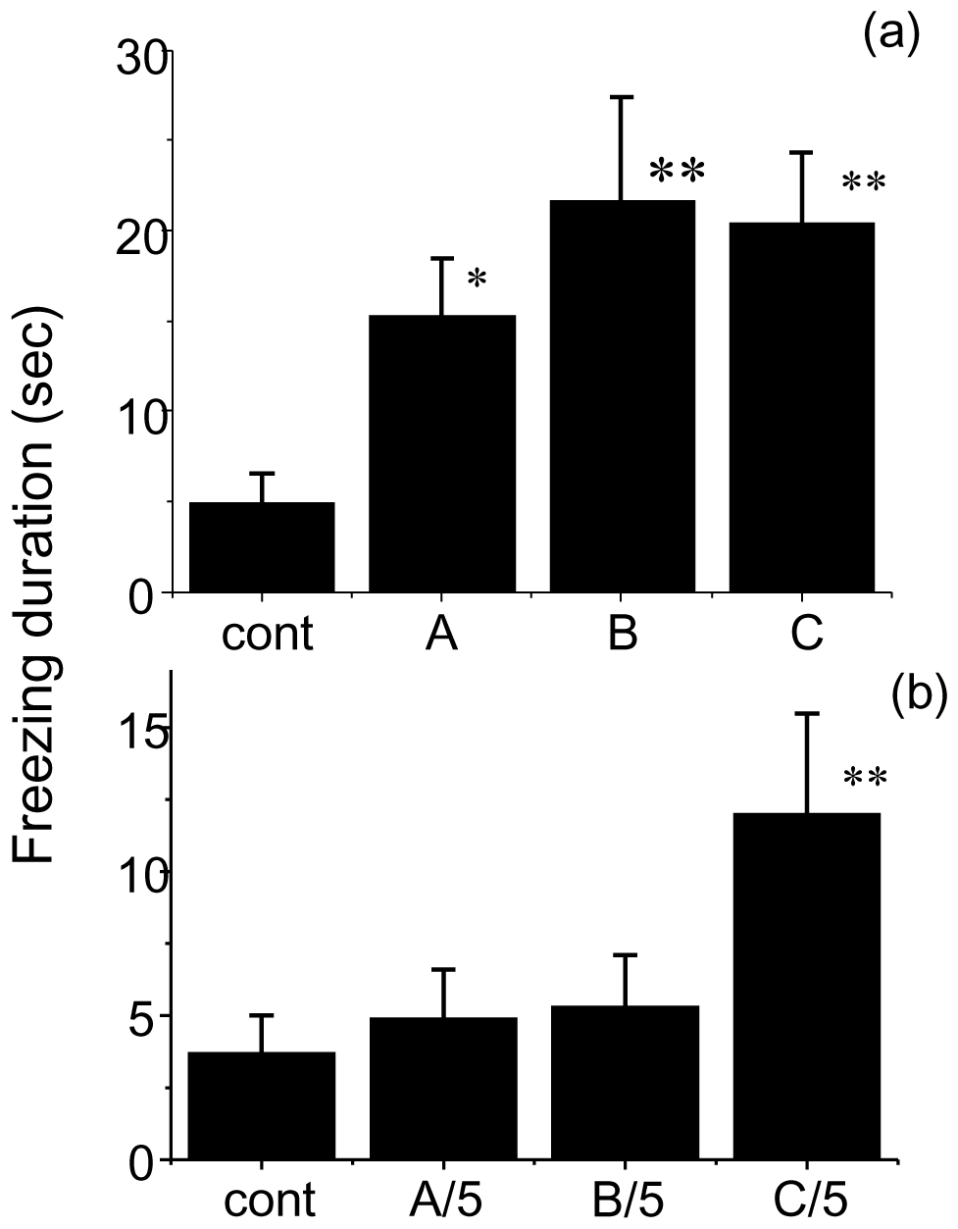
Comparison of freezing (immobilization) duration in mice during a 3 min exposure to (a) undiluted wolf urine samples and (b) 5-fold diluted wolf urine samples. The statistical significance of the differences between the freezing duration in response to wolf urine samples compared with control (water) was assessed by ANOVA followed by Fisher’s PLSD post-hoc test (*P<0.05, **P<0.01 vs. control).

Next, a test for freezing (i.e., immobilization) was performed with the three wolf urine samples. Figures 2a and b show the mean time spent freezing during a 3 min exposure to wolf urine. An overall ANOVA with odour as a between-subject factor revealed a significant main effect of wolf urine odour on freezing behaviour (undiluted urine samples, F(3,44) = 4.006, P<0.05; 5-fold diluted samples, F(3,41) = 3.400, P<0.05). Dunnett's post-hoc test showed that all three wolf urine samples induced significantly more freezing behaviour than the water control (P<0.05 for sample A, and P<0.01 for samples B and C). On the other hand, when the samples were diluted 5-fold, only sample C yielded freezing behaviour that was significantly different from that elicited by the water control (P<0.01; Figure 2b).

### (b) Fos Immunoreactivity in the AOB after Exposure to Wolf Urine Samples

**Figure 3:**
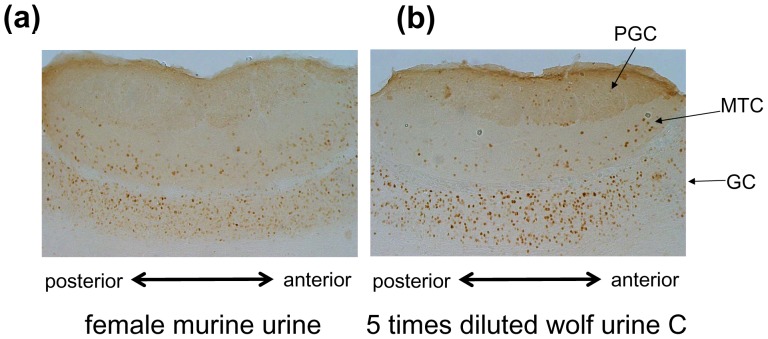
Sagittal section of the AOB of female mice stained with an antibody against Fos after exposure to female murine urine (A) or 5-fold diluted wolf urine sample C (B). PTC, periglomerular cell layer; MTC, mitral cell layer; GC, granule cell layer.

To explore the signalling pathway elicited by wolf urine odorants, we examined immunoreactivity for Fos, a marker of neuronal excitation, in the main olfactory bulb (MOB) and the AOB. Figure 3 shows that Fos immunoreactivity was particularly intense in the AOB after exposure to the 5-fold diluted wolf urine. Therefore, we focused on the AOB in the following immunohistochemical investigations.

Exposure of the vomeronasal organ (VNO) of female mice to urine excreted from female mice induced moderate Fos expression in the AOB (Figure 3A), while exposure to the 5-fold diluted wolf urine sample C resulted in stronger Fos expression (Figure 3B). After exposure to the 5-fold diluted wolf urine, Fos-immunoreactive cells were found in the posterior granule cell (PGC) layer, the mitral cell (MTC) layer and the granule cell (GC) layer. The greatest density of labelled cells was observed in the GC layer. These results suggest that substances contained in wolf urine provoke excitation of the vomeronasal system.

**Figure 4:**
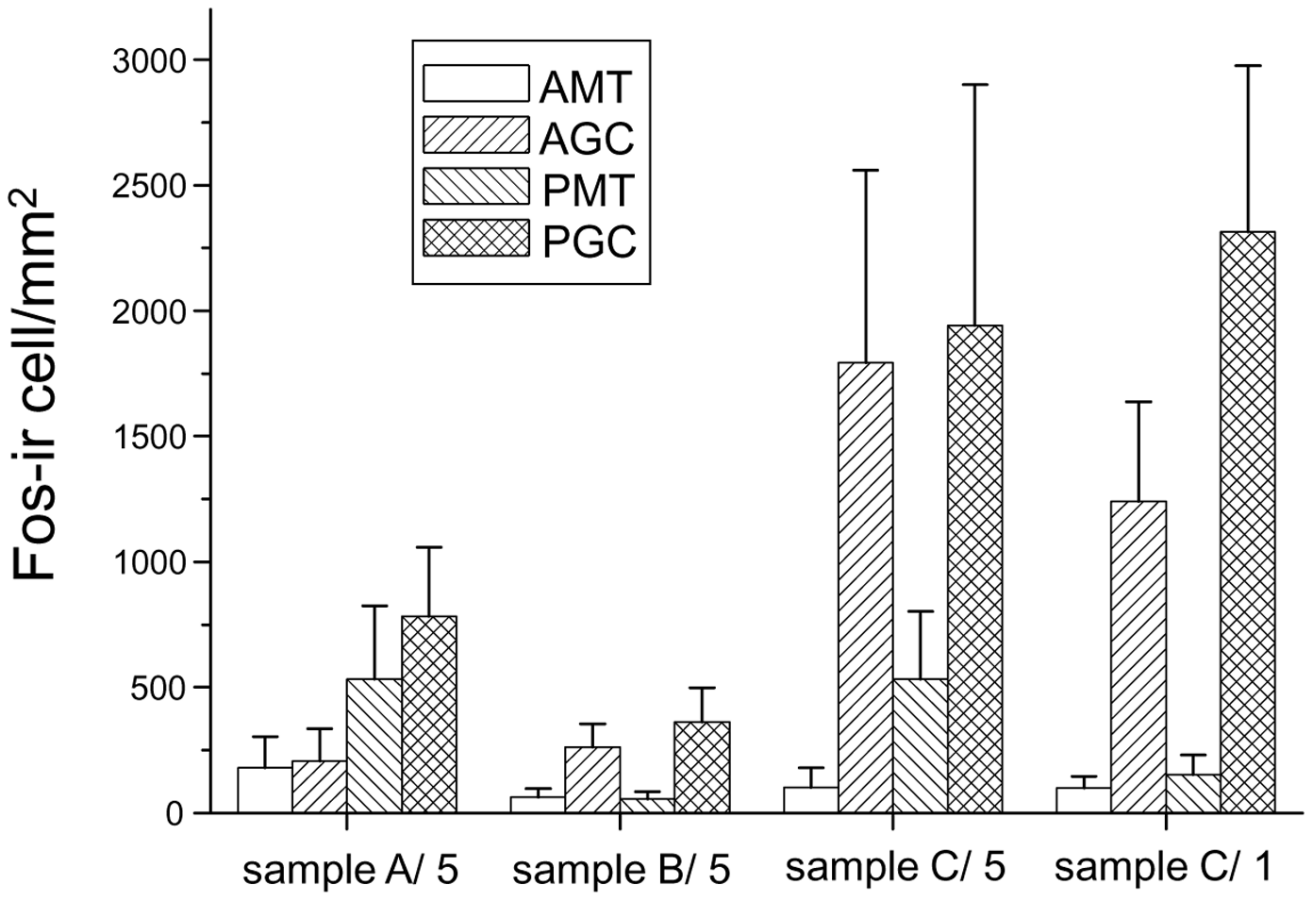
Density of Fos-immunoreactive cells (number of cells/mm^2^) in the MTC and GC layers in the anterior and posterior regions of the AOB after exposure to undiluted wolf urine sample C (sample C/1) or 5-fold diluted samples A, B or C (sample A/5, sample B/5, sample C/5). The statistical significance of the differences between sample C vs. samples A and B was assessed by ANOVA followed by Fisher’s PLSD post-hoc test. The density of Fos-immunoreactive (Fos-ir) cells in the anterior GC layer was higher after exposure to diluted sample C relative to diluted sample A (P<0.01) or diluted sample B (P<0.01); similarly, the density of Fos-immunoreactive cells in the posterior GC layer was higher after exposure to diluted sample C vs. diluted sample A (P<0.05) or diluted sample B (P<0.01). Furthermore, the density of Fos-immunoreactive cells was higher after exposure to undiluted sample C vs. diluted sample A (P<0.01) or diluted sample B (P<0.005). AMT and PMT, anterior and posterior mitral cell layer; AGC and PGC, anterior and posterior granule cell layer.

Figure 4 shows the density of Fos-immunoreactive cells (number/mm^2^) in serial sagittal sections of the AOB of female mice after exposure to various wolf urine samples. The data from each group were analysed via two-factor ANOVA with urine samples and regions (anterior MTC (AMT) layer, posterior MTC (PMT) layer, anterior GC (AGC) layer and PGC layer) as independent factors. This analysis revealed a significant main effect of urine samples on overall cell density in the AOB (F (3, 32) = 4.877, P<0.01). Fisher’s PLSD post-hoc test indicated that the density of Fos-immunoreactive cells was higher after exposure to diluted sample C than that after exposure to diluted sample A (P<0.05) or diluted sample B (P<0.005). The test also indicated that the density of Fos-immunoreactive cells was higher after exposure to undiluted sample C than that after exposure to diluted sample B (P<0.01).

### (c) Chemical Studies

**Figure 5:**
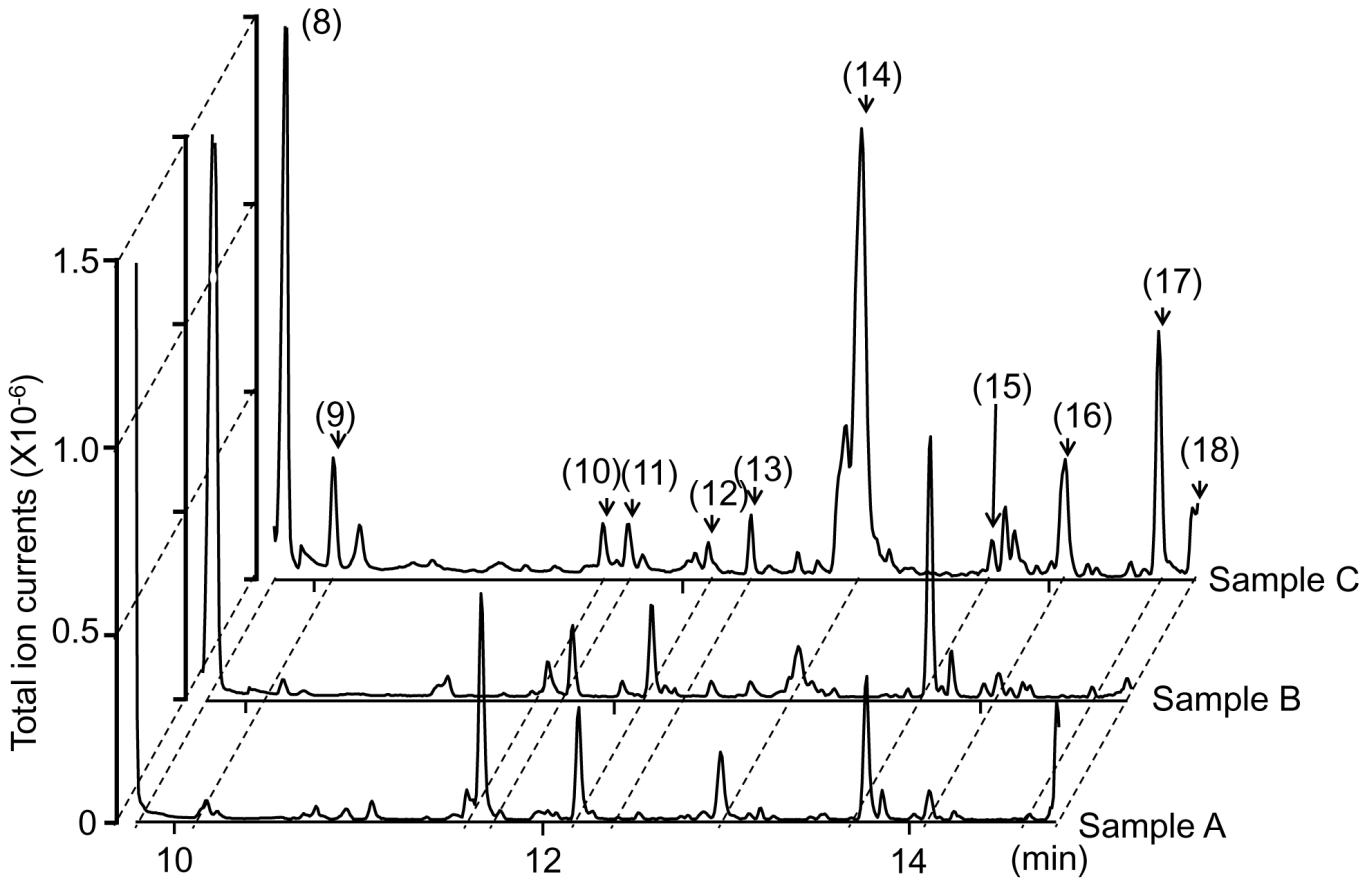
Chromatograms from GC-MS analyses of wolf urine samples. Numbers refer to the following compounds: (8) Δ^3^-isopentenyl methyl sulphide; (9) 1-(methylthio)-2-methylbut-2-ene; (10) 3-buten-1-ol, 3-methyl-; (11) 4-methyl-3-heptanone; (12) 2,4-dithiapentane*; (13) 1-pentanol, 2-methyl-; (14) pyrazine, 2,6-dimethyl- (DMP)*; (15) dimethyl trisulphide*; (16) pyrazine, trimethyl- (TMP)*; (17) pyrazine, 3-ethyl-2,5-dimethyl (EDMP)*; (18) acetic acid*. *Identified by GC-MS (n = 6) and a comparison with the retention times of identified chemicals.

Typical examples of the GC-MS chromatograms of the three wolf urine samples are shown in Figure 5. Over 50 representative peaks were chosen for comparison because we could reliably detect these peaks in at least two of the three samples. Of these, 34 were positively or tentatively identified by GC-quadropole MS (Table 1, Figure 5). Methyl isopentyl sulphide (peak #6), Δ^3^-isopentenyl methyl sulphide (peak #8), 4-methyl-3-heptanone (peak #11), etc., were previously identified in a report detailing the volatile constituents of wolf urine [10]. However, these chemicals were not enriched in sample C relative to samples A and B. Instead, sample C differed significantly from samples A and B in terms of nine peak areas that were higher in sample C and three peak areas that were lower (Table 1). DMP (2,6-dimethylpyrazine), TMP (trimethylpyrazine) and EDMP (3-ethyl-2,5-dimethyl pyrazine) were among the nine compounds that were found at high levels in sample C (Table 1). The concentrations (mean±SD) of these chemicals in urine sample C were as follows: DMP, 3.0±1.3 ppm; TMP, 0.6±0.1 ppm, DEMP, 0.9±0.2 ppm.

**Table 1:** Analysis of peak areas (as a per cent of the total) for 35 representative constituents of wolf urine samples A, B and C.

			Sample A		Sample B		Sample C			
ID#	Peak identity	RT	average	Interquartilerange	average	Interquartilerange	average	interquartile range	p<0.05	
1	Dimethyl sulfide*	1.8	0.00	0.00–0.00	3.25	3.03–3.58	1.48	1.30–1.61		
2	acetone*	2.4	0.52	0.36–0.93	0.24	0.13–0.35	0.00	0.00–0.01		
3	Methyl propyl sulfide	3.9	0.15	0.01–0.37	8.27	7.43–9.06	0.19	0.05–0.27		
4	Methyl butyl sulfide	6.6	0.09	0.06–0.12	2.01	1.57–2.77	0.07	0.06–0.09		
5	1-Propanol/Toluene*	7.2	0.27	0.10–0.58	0.43	0.06–0.74	9.07	5.94–18.06	†	
6	Methyl isopentyl sulfide*	7.9	0.07	0.05–0.12	0.30	0.19–0.41	0.08	0.07–0.10		
7	Disulfide, dimethyl*	8.0	1.71	1.52–2.13	0.54	0.39–0.74	0.05	0.01–0.11	#	
8	Δ3-Isopentenyl methyl sulfide	9.8	16.75	9.79–22.84	9.84	7.20–14.49	6.22	3.85–8.89		
9	1-(Methylthio)-2-methylbut-2-ene	10.1	0.22	0.02–0.74	0.12	0.04–0.19	0.52	0.37–0.63		
10	3-Buten-1-ol, 3-methyl-	11.7	1.17	0.96–1.67	0.62	0.51–0.83	0.29	0.16–0.45	#	
11	4-methyl-3-heptanone	11.8	0.22	0.00–0.86	0.00	0.00–0.00	0.00	0.00–0.01		
12	2,4-Dithiapentane*	12.2	0.52	0.41–0.71	0.99	0.81–1.19	0.14	0.12–0.15	#	
13	1-Pentanol, 2-methyl-	12.4	0.03	0.00–0.10	0.13	0.00–0.31	0.21	0.17–0.23		
14	Pyrazine, 2,6-dimethyl-*	13.0	0.68	0.33–1.64	1.09	1.00–1.21	4.16	3.56–4.68	†	
15	Dimethyl trisulfide*	13.7	1.26	0.66–2.39	2.52	2.25–3.08	0.08	0.01–0.13		
16	Pyrazine, trimethyl-*	14.1	0.19	0.13–0.34	0.34	0.32–0.36	0.66	0.58–0.79	†	
17	Pyrazine, 3-ethyl-2,5-dimethyl-*	14.6	0.04	0.02–0.09	0.09	0.07–0.10	1.04	0.95–1.16	†	
18	Acetic acid*	14.7	1.04	0.17–2.28	2.70	0.11–5.04	0.01	0.01–0.01		
19	1-Hexanol, 2-ethyl-	15.1	0.20	0.00–0.47	1.03	0.79–1.35	5.28	0.39–10.44		
20	Geranyl nitrile	15.2	12.41	10.64–13.67	10.29	7.31–14.11	9.34	5.64–11.55		
21	Unknown	15.3	0.00	0.00–0.00	0.03	0.00–0.08	2.17	1.74–2.59	†	
22	Propanoic acid*	15.9	0.09	0.00–0.19	0.17	0.02–0.29	0.01	0.01–0.01		
23	Acetophenone*	17.5	1.40	0.94–1.99	1.19	0.97–1.62	5.84	5.48–6.49	†	
24	Oxirane, [(1-methylethoxy)methyl]-	18.3	0.80	0.45–1.64	2.62	0.12–5.63	0.62	0.34–0.93		
25	Acetamide*	18.4	0.29	0.09–0.64	0.02	0.00–0.05	0.00	0.00–0.00		
26	Aniline*	18.7	0.00	0.00–0.01	0.15	0.02–0.24	0.75	0.65–0.85		
27	Benzenemethanol	19.3	0.85	0.66–1.37	0.68	0.64–0.73	2.49	2.11–2.69	†	
28	4,6-Dimethyl-[1], [2], [3]trithiane	19.9	5.24	0.00–12.34	7.80	3.30–13.31	7.36	1.80–11.70		
29	Quinoline, 2-methyl-*	21.2	11.62	0.96–16.28	12.42	10.81–15.92	14.52	13.48–17.00		
30	Phenol*	21.4	20.41	18.35–23.35	22.01	19.78–23.94	10.81	9.95–11.75		
31	Phenol, 4-methyl-*	22.2	1.18	0.74–2.28	3.69	2.59–4.46	7.64	6.92–8.85	†	
32	Phenol, 4-ethyl-*	23.3	0.00	0.00–0.00	0.01	0.00–0.05	2.08	1.71–2.72	†	
33	Benzoic acid*	26.8	0.24	0.12–0.39	0.02	0.00–0.04	0.00	0.00–0.00		
34	Indole*	27.0	19.32	17.84–20.60	4.34	3.36–5.78	5.95	1.92–8.57		

The median area is shown for six MS experiments. *Positively identified by GC-quadropole MS and by matching the retention times against identified chemicals in wolf urine. The chemicals without symbols were tentatively rather than positively identified by GC-quadropole MS. ^†^Signifies a significantly higher peak area, whereas #signifies a significantly lower peak area, in sample C compared with either sample A or B, as assessed by the Kruskal-Wallis test and the Steel-Dwass post-hoc test.

Although 2,5-dimethylpyrazine (not 2,6-dimethylpyrazine) has been reported to be a mouse pheromone and has been used in previous studies [20], [21], there is little evidence of the presence of DMP, TMP or EDMP in mouse urine [22]–[28], and only one study has detected trace levels of DMP and TMP in mouse urine [29]. In addition, although these volatile pyrazines are characterized by a strong odour, they do not appear to facilitate conspecific communication among canines [10], [12]. Therefore, it is conceivable that DMP, TMP and EDMP are novel fear-inducing kairomones that are unique to the urine of predators, including wolves. On the other hand, acetophenone, 4-methyl phenol, 4-ethyl phenol and propanol (also found at high levels in wolf urine sample C, Table 1) exist ubiquitously in the urine of all mammalian species [22]–[28], and there is no evidence to suggest that they play a role in chemosignalling [30], [31]. Therefore, we next conducted additional behavioural and immunohistochemical studies specifically for DMP, TMP and EDMP.

### (d) Behavioural Studies for DMP, TMP and EDMP

**Figure 6:**
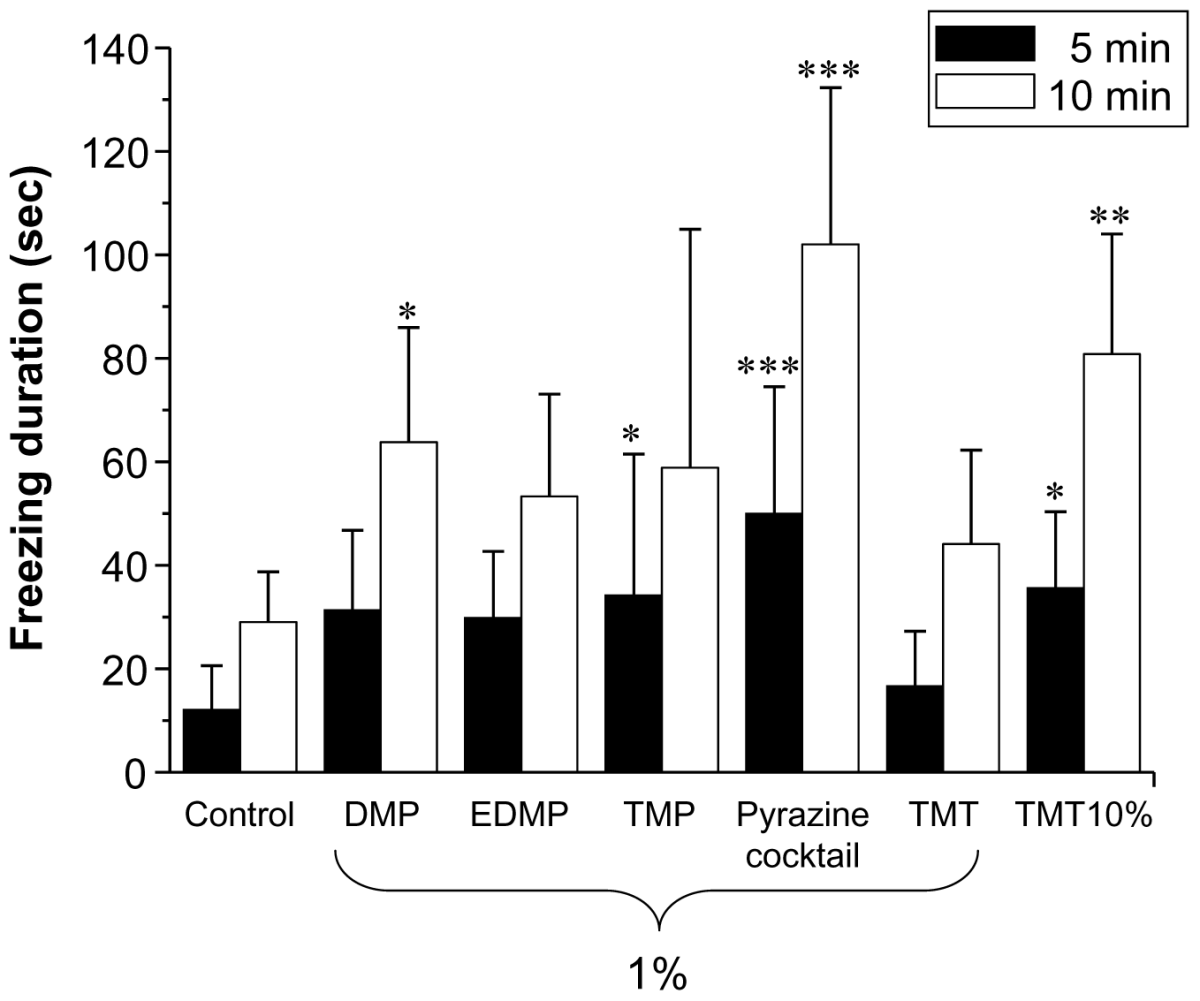
Comparison of freezing duration in mice during a 5 or 10 min exposure to pyrazine compounds. The statistical significance of the differences between the freezing duration in response to pyrazines vs. water (control) was assessed by ANOVA followed by Fisher’s PLSD post-hoc test (*P<0.05, **P<0.01, ***P<0.001 vs. control).

Figure 6 shows the mean time spent freezing during exposure to purified DMP, TMP or EDMP (50 µl of each, 1% v/v), or to a mixture of DMP, TMP and EDMP (pyrazine cocktail; 50 µl total, 0.33% v/v of each). An overall ANOVA with odour as a between-subject factor revealed a significant effect of odour on freezing behaviour [F(6,38) = 3.688, P<0.01 for a 5 min observation period, F(6,38) = 6.096, P<0.001 for a 10 min observation period]. Dunnett's post-hoc test also showed that these chemicals induced significantly more freezing behaviour than water [P<0.05 for DMP (10 min observation period) and TMP (5 min observation period); P<0.001 for the pyrazine cocktail (5 and 10 min observations periods)]. Moreover, the pyrazine-mediated freezing effects were stronger than those instigated by 2,5-dihydro-2,4,5-trimethylthiazoline (TMT; 1%), a fear-inducing stimulus identified in fox faeces [19], [32]. Furthermore, the mixture of TMP, DMP and EDMP was more effective than any pyrazine by itself and, indeed, was more efficacious than 10% TMT.

### (e) Fos Immunoreactivity in the AOB after Exposure to Urine-derived Chemicals

**Figure 7:**
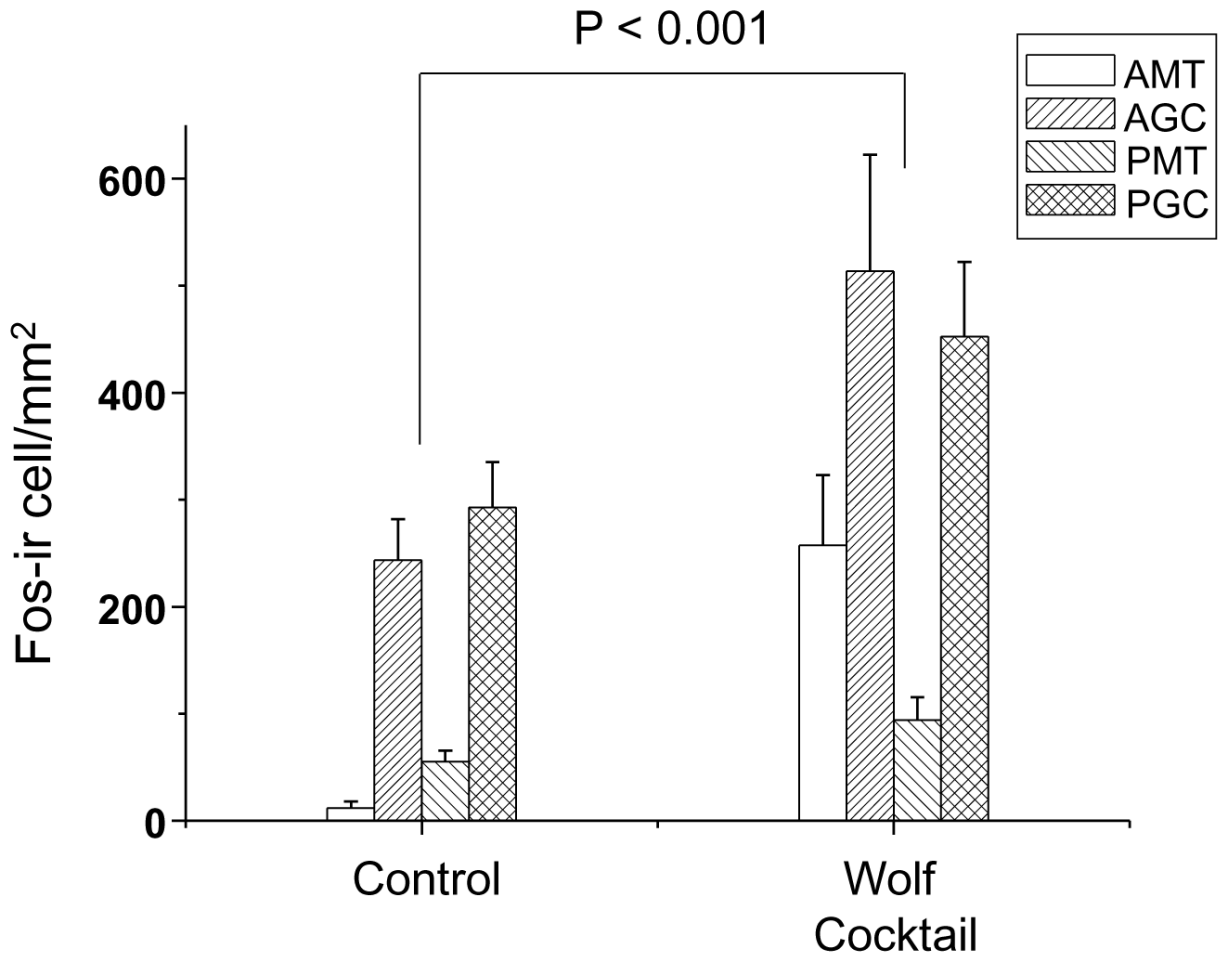
Density of Fos-immunoreactive cells (number of cells/mm^2^) in the GC and MTC layers in the anterior and posterior regions of the AOB after exposure to water or pyrazine cocktail. The density of Fos-immunoreactive (Fos-ir) cells in the AMT, AGC and PGC layers was higher after exposure to the pyrazine cocktail vs. the water control (P<0.001).

Figure 7 shows the density of Fos-immunoreactive cells (number/mm^2^) in the AOB of female mice after exposure to the pyrazine cocktail or the control (water). The data from each group were analysed by two-factor ANOVA, with the samples and investigated regions (AMT, PMT, AGC and PGC layers) as independent factors. This analysis revealed a significant main effect of sample [F (2, 68) = 25.302, P<0.001], but not of region. Fisher’s PLSD post-hoc test indicated that the density of Fos-immunoreactive cells in the AOB after exposure to the pyrazine cocktail was higher than that after exposure to water (P<0.001).

## Discussion

The present study shows that wolf urine odours are aversive and fear-inducing to female mice (Figures 1, 2) and that this is due, at least in part, to changes in the levels of specific volatile urinary compounds (Table 1, Figure 5). To the best of our knowledge, this is the first investigation to show that wolf urine can evoke fear in mice.

This study was conducted with three different wolf urine samples, A, B and C. The results of our behavioural experiments indicate that there are significant differences in the fear-inducing effects between wolf urine sample C and samples A and B (Figures 1 and 2), with sample C having the most pronounced activity. Moreover, immunohistochemical analyses indicated that exposure to sample C significantly increased the density of Fos-immunoreactive cells in the murine AOB compared with murine urine (Figure 3) or samples A and B (Figure 4). Furthermore, chemical analyses revealed that the levels of pyrazine analogues, namely, DMP, TMP and EDMP, were significantly higher in sample C relative to samples A and B (Table 1, Figure 5). Notably, the pyrazine analogues mimicked the ability of wolf urine to induce freezing behaviour (Figure 6), especially when combined in a pyrazine cocktail. Therefore, our present study indicated that pyrazine chemicals identified in wolf urine comprise a set of novel kairomones that initiate defensive behaviour in mice.

Other predator-derived odorants also elicit powerful aversion and freezing responses in the rodent. For example, naïve rats and mice exposed to the odour of foxes or TMT [16], [32], the most effective fear-inducing component in fox faeces, showed species-specific defensive responses, such as freezing-in-place [17]–[19], [33]. Similarly, rodents exposed to cat-derived odours demonstrated dose-dependent fear-related responses, including freezing, avoidance and the elevation of stress hormones [34]–[36]. In addition, Ferrero et al. reported that 2-phenylethylamine (2-PEA), a common constituent of carnivore urine (including lion, serval, tiger and jaguar urine), is a key component of an odorant blend that triggers hard-wired aversion circuits in the rodent brain [15]. In the present study, we did not identify 2-phenylethylamine as an avoidance odour chemical. This discrepancy is likely due to differences in the analytical methods used, namely head space solid phase micro-extraction (HS-SPME) and extraction with an organic solvent. In some cases, these two sampling approaches are complementary and can generate different results [37], [38].

The chemicals in the above studies [25]–[33] exerted their actions by stimulating the main olfactory system. Although the pyrazine volatiles identified in the current study primarily affected Fos immunoreactivity in the murine AOB, it is conceivable that they also stimulated the MOB to evoke freezing behaviour. DMP, TMP and EDMP are all volatile chemicals that emit a pungent odour, and freezing behaviour is only observed in response to repugnant predator odours, such as TMT. Notably, the activity of the pyrazine cocktail (0.33% v/v of each) was stronger than that of 10% TMT, suggesting that the putative wolf urine-derived pyrazine kairomones have a more pronounced fear-inducing effect than TMT, at least in mice. However, TMT is more effective in inducing fear in rats than mice [19], [39], [40]; therefore, further investigation is needed to clarify how these wolf urine-derived pyrazine kairomones induce fear in rats.

As noted above, the pyrazine cocktail, like wolf urine sample C, increased Fos expression in the AOB of female mice (Figure 7). The rodent vomeronasal system plays a crucial role in mediating pheromone-evoked social and sexual behaviours [41]. Recent studies demonstrated that the VNO and its synaptic target, the AOB, are involved not only in conspecific pheromone perception, but also in the interspecific detection of kairomones. For example, Ben-Shaul et al. [42] identified a significant fraction of murine AOB neurons that responded robustly and selectively to predator cues. In addition, the exposure of rodents to cat odours augmented the number of Fos-positive cells in the AOB [43].

Until recently, the chemical basis for AOB stimulation by cat odours remained elusive. However, Papes et al. [44] demonstrated that rat lipocalin encoded by the Mup 13 gene and recombinant feline Mup (based on Mup Feld4 in cat saliva) are sufficient to activate VNO and AOB neurons and initiate defensive behaviour (but not freezing behaviour) in mice. In addition, cat odour alone activates the rat AOB and its projection areas in the extended amygdala, as well as a medial hypothalamic circuit that is strongly associated with defensive behaviour [43]. On the other hand, little is understood in terms of chemical mechanisms behind cat odour-induced freezing behaviour. In this regard, the present observations are the first to identify volatile urinary chemosignals (i.e., pyrazine analogues) that provoke fear-associated immobilization and stimulate the rodent AOB, and perhaps the MOB as well.

The mechanism(s) that might underlie the formation of pyrazine analogues in wolf urine is unknown. However, an intriguing possible explanation is related to the glycation which occurs in living animals [45], [46]. Alkylpyrazines are a typical class of glycation compound [47], and TMP and DMP are formed between glycine oligopeptides and reducing sugars [48]. Importantly, food-derived oligopeptides are detected in the blood flow after oral ingestion of meat and collagen [49], [50]. Therefore, high amounts of amino compounds in prey or foods containing meat or connective tissue may be the source from which pyrazine analogues are generated in carnivores (including wolf).

Our observations also raise the question of why sample C contains salient amounts of pyrazine analogues. A likely explanation is related to the harvest season of the urine samples. Raymer et al. [10] reported that several wolf urine-derived compounds displayed seasonal dependence, with levels of certain compounds increasing in March, one month after the conclusion of the breeding season. Sample C was harvested in March, whereas samples A and B were harvested in November and January, respectively. This suggests that pyrazine analogue levels may also increase in the urine near the end of the breeding season.

Alternatively, harvest season-associated changes in food quantity or quality may affect the urinary content of pyrazine analogues; indeed, considerable evidence indicates that short-term fluctuations in urinary content and odour can result from dietary changes [28], [51], [52]. Moreover, Nolte et al. [11] demonstrated that diet composition and sulphurous metabolites of meat digestion are important factors for the repellency of predator odours against potential prey. Therefore, seasonal changes in food quality could account for alterations in pyrazine analogue concentrations in the urine of donor wolves. Finally, wolf urine constituents can be modified by gender and social status [53]. In our study, the influences of gender and social hierarchy within the pack cannot be excluded. Clearly, further investigation is needed to clarify why pyrazine levels are elevated in certain wolf urine samples.

In conclusion, our findings clearly illustrate the following points. 1) Wolf urine odours induce aversive and fear-related responses in female mice. 2) These activities are mainly due to the presence of particular volatile pyrazine compounds, namely, DMP, TMP and EDMP, in wolf urine. 3) The mixture of DMP, TMP and EDMP is more potent than any one component alone. 4) Wolf urine and the pyrazine mixture both stimulate the murine vomeronasal system.

Regarding this final point, we note that although wolf urine has been put to practical use for the eradication of wild animals from residential areas, pyrazine analogues are readily available and hence more convenient for this application than wolf urine. However, further studies are required to confirm whether pyrazine analogues can mimic the dispersive actions of wolf urine. Moreover, the current discovery that pyrazine analogues provoke repugnance in mice provides a strong rationale for additional studies of odorant-induced behaviours and the neurophysiological mechanisms thereof.

## Materials and Methods

### (a) Animals

The animals were cared for in accordance with the National Institutes of Health (NIH) Guide for the Care and Use of Laboratory Animals. The Animal Ethics and Research Committee of the Health Sciences University of Hokkaido approved the experimental protocols prior to the initiation of the study (approval ID: 035).

Experimental animals were female C57BL/6J mice, unless otherwise stated [19]. Male mice showed identical responses to female mice as analyzed by c-Fos expression and behaviour (data not shown). Animals were upheld in a room maintained at 22°C with a photoperiod of 12 h:12 h (non-reversed 12 h light/dark cycle). The mice were housed in polycarbonate cages (two to three animals per cage) in a sterile animal facility. They were also provided with ad libitum access to a standard murine diet (Lab Chow, MF, Oriental Yeast, Tokyo, Japan) and water.

### (b) Wolf urine Samples

Wolf urine samples were purchased from PredatorPee, Inc. (Bangor, ME, USA). The urine samples harvested from both male and female wolves (n >10) belonging to the same pack. The urine was collected via floor collection drains in cages, and the animals were consistently treated in the most humane manner possible. The urine samples were harvested on average 3–4 weeks before sale [54]. The three different wolf urine samples (samples A, B and C; six bottles of each) employed in this study were purchased in December 2009, February 2010 and April 2010, shortly after their harvest. Therefore, the approximate harvest dates were November 2009 (sample A), January 2010 (Sample B) and March 2010 (sample C). Because wolves consume much more water in a hot environment, which then dilutes the urine, urine samples collected during the summer months were not employed in this study. The urine samples were stored in sterile tubes at −20°C until use; they were then employed as undiluted samples, or alternatively, they were diluted with MilliQ water just before use.

### (c) Overall Design of GC-MS Experiments

The differences between the three wolf urine samples were determined in terms of their volatile components by using GC-MS analysis. The peak areas of 34 representative compounds present in each of the three samples were investigated.

### (d) Preparation of Samples for GC-MS Analysis by Head Space Solid Phase Micro-extraction (HS-SPME)

Samples were prepared for GC-MS analysis by using HS-SPME. To concentrate the volatiles in each urine sample, a SPME fibre (2 cm, 50/30 µm divinylbenzene (DVB)/carboxen/polydimethylsiloxane (PDMS) StableFlex fiber; Supelco, Bellefonte, PA, USA) was inserted for 30 min into the head space of a 4 ml vial with a Teflon septum (Supelco) containing wolf urine (150 µl). The urine sample was then saturated with NaCl and mildly heated to 37–40°C with constant stirring.

### (e) Chemical Analysis Performed with GC-MS

The identification of odorants was performed by using a GC-quadropole MS PARVUM 2 System (Shimadzu Corporation, Kyoto, Japan). The gas chromatograph was fitted with a Restek Stabilwax column (30 m×0.32 µm×0.5 µm; Restek, Bellefonte, PA, USA). Helium was used as the carrier gas, and the column flow was 2.4 ml/min. The oven temperature was maintained at 40°C for 5 min, increased by 10°C/min to 200°C, and finally elevated by 5°C/min to 240°C. The injector temperature was held constant at 230°C.

The identification of structures corresponding to the representative peaks was performed by using both the National Institute of Standards and Technology (NIST) Mass Spectral 08 Library and a manual interpretation of the mass spectra based on comparisons with those reported in the literature [10]. In addition, a comparison was performed with the relative retention times and mass spectra of purified DMP, TMP and EDMP. These pyrazines were kindly supplied by Takasago International Corporation (Tokyo, Japan) and preserved as glycerol triacetate solutions (1% v/v) in a deep freezer until use.

The statistical significance of differences between samples A, B and C in terms of chemical constituents was assessed by the Kruskal-Wallis test, followed by the Steel-Dwass post-hoc test (see Table 1).

### (f) Avoidance Behaviour in Response to Wolf Urine Samples

Twenty-four sexually inexperienced 2- to 5-month-old mice served as the test animals. Test mice were assigned to one of the three wolf urine sample groups (A, B or C) or to the control group (water) (n = 6 per group). The mice were employed in three separate avoidance tests involving exposure to 1) undiluted, 2) 5-fold diluted and 3) 15-fold diluted urine. The tests were conducted once per day, and each series of tests was conducted one week after the previous series. The mice were confronted with the urine samples in descending order of concentration.

The aversion tests were conducted in a Y maze. A custom-made Y maze (long arm length, 450 mm; short arm length, 400 mm; arm width, 100 mm) was constructed from Plexiglas (1 cm thick). The design of the Y maze used in this study is described in detail elsewhere [52], [55]. Before the initiation of the experiment, the mice were habituated to the Y maze for 3 min. The next day, bedding material (15 g) was mixed with wolf urine (10 ml), placed into a plastic dish (7×7 cm), and inserted into the odour box at the back of one of the short arms. The same amount of bedding was mixed with water (control, 10 ml) and inserted into the odour box at the back of the other short arm. Next, the amount of time that the mouse spent in each of short arms of the Y maze, which were directly connected to the odour boxes, was measured over a period of 3 min. The odour sources were inserted at random into one odour box or the other adjoined to the short arms of the Y maze.

Each test was conducted between 12∶00 noon and 17∶00. The floor of the test area was replaced with a clean bench coat between each trial to eliminate residual odorant cues. All behavioural studies were performed as blind tests. To demonstrate the avoidance of wolf urine samples by the mice, the behavioural data were processed as follows. The avoidance rate was taken to be the amount of time spent in the short arm with the control odour divided by the total amount of time spent in the both short arms(control vs. wolf urine), then multiplied it by 100. Water was substituted for wolf urine in control experiments (control vs. control). The statistical significance of the differences between the avoidance rates elicited by each wolf urine sample was assessed by ANOVA followed by Fisher’s PLSD post-hoc test. Data are given as the means ± the SEM.

### (g) Freezing Behaviour in Response to Wolf Urine Samples

Sexually inexperienced 2- to 5-month-old mice (n = 132) served as the test animals. To explore the fear-inducing properties of the wolf urine samples and the purified pyrazines, freezing-in-place tests were conducted in a rectangular, open-bottomed polycarbonate chamber (27 cm × 17 cm × 14 cm). An aluminium exhaust duct (11 cm in diameter) attached to the polyethylene mesh on the inlet was connected from the upper centre of the chamber to the exhaust port in the experimental room.

The mice were first familiarized to the chamber by placing them inside for 5 min on each of two consecutive days. On the third day, 15 g of bedding was mixed with either 10 ml of undiluted urine sample, 5-fold diluted urine sample or water (control). The beddings were placed into plastic dishes (7×7 cm) and inserted into the chamber. Alternatively, individual pyrazines (DMP, TMP and EDMP) identified in the wolf urine samples (50 µL of each, 1% v/v) or a mixture of DMP, TMP and EDMP (50 µL total volume, 0.33% v/v of each component) were applied to pieces of filter paper (2×2 cm), placed into Petri dishes, and inserted into the chamber. The freezing duration was compared with that induced by an identified fear-inducing compound in fox faeces, TMT (1% and 10%) [16]–[18], [32]. To avoid confounding the data due to learning, the female mice were only tested once.

The statistical significance of differences between odour samples was assessed by ANOVA followed by Fisher’s PLSD post-hoc test. Data are given as the means ± the SEM.

### (h) Tissue Processing and Immunohistochemistry

Adult mice (> than 6 months old; n = 27) were exposed for 90 min to urine samples or pyrazine cocktail (5 ml of each, applied with a cosmetic atomizer) in the form of paper-soiled bedding (Japan SLC, Hamamatsu, Japan). The animals were then deeply anaesthetized with pentobarbital sodium (35 mg/kg) at 75 min after exposure to the urine samples or the pyrazine stimulus. The animals were perfused through the heart with phosphate-buffered saline (PBS), followed by fixation with 4% paraformaldehyde. The olfactory bulbs including the AOB, still connected to the brain, were removed, and the tissue samples were soaked in PBS/4% paraformaldehyde fixative overnight. They were then were sliced into serial sections on a vibratome at a thickness of 100 µm. The free-floating sagittal sections were first treated with 0.3% hydrogen peroxide (H_2_O_2_) for 15 min in PBS with 0.4% Triton X-100 (PBSx), followed by two washes with PBS. After incubation for 1 h in 3% normal goat serum, the sections were incubated overnight at room temperature with anti-Fos polyclonal antibody (1∶8000, Ab-5; Calbiochem, La Jolla, CA, USA) in PBSx. The sections treated in this manner were then rinsed with PBSx and incubated with biotinylated goat anti-rabbit IgG (1∶200; Vector, Burlingame, CA) for 1 h. The sections were again rinsed with PBSx, incubated with ABC reagent (ABC Elite kit, Vector) for 1 h, and developed with DAB/H_2_O_2_ (0.05% DAB and 0.003% H_2_O_2_ in 0.05 M Tris-HCl buffer) for 12 min. The sections were rinsed with water and mounted.

The Fos-immunoreactive cells in the AOB were counted under a microscope by an individual blinded to the experimental protocol. The areas corresponding to the MTC and GC layers were measured in photographs taken of all stained sections of the AOB by using SigmaScan Pro software (SPSS Inc., Chicago, IL, USA). Three sections through larger areas of the AOB were employed to calculate the average density of the Fos-immunoreactive cells in the AOB of individual mice. Differences in the density of Fos-immunoreactive cells as a function of urine sample were analysed by ANOVA followed by Fisher’s PLSD post-hoc test. Data are given as the means ± the SEM.
